# High-Throughput Sequencing Reveals Three Rhabdoviruses Persisting in the IRE/CTVM19 Cell Line

**DOI:** 10.3390/v16040576

**Published:** 2024-04-09

**Authors:** Alexander G. Litov, Alexey M. Shchetinin, Ivan S. Kholodilov, Oxana A. Belova, Magomed N. Gadzhikurbanov, Anna Y. Ivannikova, Anastasia A. Kovpak, Vladimir A. Gushchin, Galina G. Karganova

**Affiliations:** 1 Laboratory of Biology of Arboviruses, FSASI “Chumakov FSC R&D IBP RAS” (Institute of Poliomyelitis), 108819 Moscow, Russia; novosti-wxo@yandex.ru (A.G.L.); ivan-kholodilov@bk.ru (I.S.K.); mikasusha@bk.ru (O.A.B.); magomed_19@mail.ru (M.N.G.);; 2 Institute for Translational Medicine and Biotechnology, Sechenov University, 119991 Moscow, Russia; 3 Pathogenic Microorganisms Variability Laboratory, Gamaleya Federal Research Centre for Epidemiology and Microbiology, Ministry of Health of the Russian Federation, 123098 Moscow, Russia; shchetinin.alexey@yandex.ru (A.M.S.); wowaniada@gmail.com (V.A.G.); 4 Faculty of Biology, Lomonosov Moscow State University, 119991 Moscow, Russia; 5 Laboratory of Biochemistry, FSASI “Chumakov FSC R&D IBP RAS” (Institute of Poliomyelitis), 108819 Moscow, Russia; freiheit007@mail.ru

**Keywords:** tick cell line, IRE/CTVM19, *Rhabdoviridae*, endogenous virus, persistence, IRE/CTVM19-associated rhabdovirus, Norway mononegavirus 1, Chimay rhabdovirus

## Abstract

Cell cultures derived from ticks have become a commonly used tool for the isolation and study of tick-borne pathogens and tick biology. The IRE/CTVM19 cell line, originating from embryos of *Ixodes ricinus*, is one such line. Previously, reovirus-like particles, as well as sequences with similarity to rhabdoviruses and iflaviruses, were detected in the IRE/CTVM19 cell line, suggesting the presence of multiple persisting viruses. Subsequently, the full genome of an IRE/CTVM19-associated rhabdovirus was recovered from a cell culture during the isolation of the Alongshan virus. In the current work, we used high-throughput sequencing to describe a virome of the IRE/CTVM19 cell line. In addition to the previously detected IRE/CTVM19-associated rhabdovirus, two rhabdoviruses were detected: Chimay rhabdovirus and Norway mononegavirus 1. In the follow-up experiments, we were able to detect both positive and negative RNA strands of the IRE/CTVM19-associated rhabdovirus and Norway mononegavirus 1 in the IRE/CTVM19 cells, suggesting their active replication in the cell line. Passaging attempts in cell lines of mammalian origin failed for all three discovered rhabdoviruses.

## 1. Introduction

Ticks are hosts to a wide variety of bacteria and viruses [1,2], some of which can infect humans via tick bites and can cause disease. For example, thousands of tick-borne encephalitis cases and hundreds of thousands of Lyme borreliosis cases (caused by some *Borrelia* species) are reported annually worldwide [3,4,5].

Cell cultures derived from tick cells are an essential tool for research on tick-related pathogens. They are used for the isolation, reproduction, and study of tick-infecting viruses and bacteria [6]. A vast number of tick-borne pathogens have been studied using tick cell lines, including various flaviviruses [7], Uukuniemi virus [8], Crimean–Congo hemorrhagic fever virus (CCHFV) [9], *Rickettsia raoultii* [10], *Spiroplasma* sp., *Rickettsia slovaca*, and *Mycobacterium* sp. [11]. Recently, the scope of tick cell cultures has broadened to include studies focused on tick biology [6,12]. The IRE/CTVM19 cell line is derived from the embryos of *Ixodes ricinus* ticks [6]—the most abundant tick species in Europe [2]. This cell line has become a common tool for the study of both various tick-borne pathogens and tick biology [8,12,13,14].

Some tick cell lines have been proven to be hosts for persisting viruses. For example, an orbivirus, St. Croix River virus (SCRV), was detected in an IDE2 cell line derived from *Ixodes scapularis* [15] and in RA243 and RA257 derived from *Rhipicephalus appendiculatus* [16]. The screening of multiple tick cell lines with pan-nairovirus oligonucleotides showed a positive yield of PCR products with similarities to CCHFV; however, it was not clear whether this amplification was specific [16].

Bacterial DNA sequences have been detected in multiple tick cell lines. Sequences of the genus *Rickettsia* were detected in the *Amblyomma-variegatum*-derived AVL/CTVM13 and AVL/CTVM17 cell lines, and sequences of the genus *Francisella* were detected in the *Dermacentor albipictus* DALBE3, *Dermacentor nitens* ANE58, and *Dermacentor variabilis* DVE1 cell lines [16]. However, the presence of intracellular bacteria was not detected via transmission electron microscopy. 

Endogenous and persistent viruses can affect the properties of viruses grown in a cell line and can alter the results of studies focused on tick biology. Thus, knowledge of the virome of tick cell lines is necessary for more accurate interpretation in further studies. There have also been several reports about the presence of viruses in the IRE/CTVM19 cell line. First, reovirus-like particles were identified via electron microscopy [16]. At the same time, attempts to obtain viral sequences using pan-flavivirus, SCRV, pan-nairovirus, and CCHFV-specific oligonucleotides failed [16]. It was also reported that sequences with similarity to rhabdoviruses and iflaviruses were identified in the IRE/CTVM19 cell line; however, no genome sequences were assembled [17]. The full genome of one rhabdovirus, subsequently named IRE/CTVM19-associated rhabdovirus (IARV), was later obtained during the isolation and sequencing of the Alongshan virus [18].

Recently, thousands of novel viruses have been discovered in various arthropods using a metagenomic approach [19,20], including many tick-related viruses [21,22]. This process has led to major changes in the virus taxonomy. In the *Rhabdoviridae* family, which consists of viruses with single-stranded negative-sense RNA genomes with an N–P–M–G–L genome organization, five new genera of tick-infecting viruses have been established: *Lostrhavirus*, *Zarhavirus*, *Sawgrhavirus*, *Alpharicinrhavirus*, and *Betaricinrhavirus*. 

Here, we used such an approach to describe the virome of the IRE/CTVM19 cell line. We identified three viruses in the cell line, obtained the full-genome sequences of two of them, and studied their ability to reproduce in mammalian cell lines.

## 2. Materials and Methods

### 2.1. IRE/CTVM19 Cell Line Maintenance

Uninfected tick cell line IRE/CTVM19 [6] provided by the Tick Cell Biobank (University of Liverpool, UK) was used in this study. The cell line was maintained at 28 °C in L-15 medium (FSASI Chumakov FSC R&D IBP RAS, Moscow, Russia) supplemented with 10% tryptose phosphate broth (Difco, Detroit, MI, USA), 20% fetal bovine serum (FBS, Gibco, Paisley, UK), 2 mM L-glutamine (FSASI Chumakov FSC R&D IBP RAS, Moscow, Russia), and antibiotics (ciprofloxacin). Cell culture supernatant was collected weekly and used as material for further work.

### 2.2. High-Throughput Sequencing

Sample preparation and high-throughput sequencing were performed as described previously [18]. Spent medium (supernatant) of the IRE/CTVM19 cell culture was used. It was clarified by centrifugation using an SW-28 rotor in an Optima L-90K Ultracentrifuge (Beckman Coulter, Brea, CA, USA) at 17,984× *g* for 30 min at 4 °C, and then, it was ultracentrifuged at 112,398× *g* for 6 h at 4 °C using the same rotor. 

The total RNA was isolated from the ultracentrifugation pellet using an RNeasy Mini Kit (Qiagen, Hilden, Germany) according to the manufacturer’s protocol. It was fragmented and reverse-transcribed into cDNA with random hexamer primers using RevertAid reverse transcriptase (ThermoFisher Scientific, Waltham, MA, USA), followed by second-strand synthesis with the NEBNext Ultra II Non-Directional RNA Second-Strand Synthesis Module (NEB, Ipswich, MA, USA). The resulting double-stranded DNA was purified using Ampure XP (Beckman Coulter, Brea, CA, USA) and used as an input for the library preparation process using the NEBNext^®^ Fast DNA Library Prep Set for Ion Torrent™ (NEB, Ipswich, MA, USA) following the manufacturer’s instructions. The DNA library obtained was quantified with an Ion Library TaqMan™ Quantitation Kit, followed by templating on an Ion Chef (ThermoFisher Scientific, Waltham) instrument and sequencing on an Ion S5XL instrument, with the viral library constituting part of an Ion 530 Chip. The reads obtained were deposited in the sequence read archive database under accession number PRJNA879987.

### 2.3. Virus Genome Assembly

Raw reads were quality-controlled using FaQCs v2.09 [23] and assembled into contigs using SPAdes v3.13.0 [24] with iontorrent flag. Contigs containing homologies with known viruses were identified by comparison with an nt database using the BLAST v.2.12.0 program. 

Using only a SPAdes assembly, we were unable to obtain contigs corresponding to the full-length virus genomes. Since all of the detected viruses had very close homologs in the GenBank database, we mapped the reads on sequences from the GenBank database. Either raw reads or reads filtered by length (more than 34 nt) and quality (Q20) with Trimmomatic v. 0.39 [25] were used for mapping. Mapping was performed using UGENE v.40.0 software [26] with up to a 10% mismatch per read allowed and “align reverse complement reads” enabled. A consensus sequence was extracted from the assembly with the “extract consensus” option in the UGENE program; this consensus sequence was considered the full-genome sequence of the virus.

The percentage of virus-containing reads in the data was estimated using the Bowtie program [27]. The percentage of reads aligned exactly once on the full virus genome was considered the percentage of virus-containing reads.

### 2.4. RNA Isolation and Virus Detection

The total RNA was isolated from the cell culture supernatant using TRI Reagent LS (Sigma-Aldrich, St. Louis, MO, USA), according to the manufacturer’s protocol. 

For the passaging experiment, reverse transcription was carried out using a random hexamer oligonucleotide with the MMLV Reverse Transcriptase (Evrogen, Moscow, Russia), according to the manufacturer’s instructions. For the strand-specific PCR, reverse transcription was carried out using Chimay_L_R, Norway_L_R2, and Rhabdo_Miass_L_1R (Table S1) to target the genome (negative strand), and Chimay_L_F, Norway_L_F2 and Rhabdo_Miass_L_1F (Table S1) to target the replicative form (positive strand), using the MMLV Reverse Transcriptase (Evrogen, Moscow, Russia). A subsequent PCR was carried out with virus-specific oligonucleotides (Table S1) using the DreamTaq DNA Polymerase (Thermo Fisher Scientific, Vilnius, Lithuania) according to the manufacturer’s instructions, with DEPC-treated water used as a negative control. Oligonucleotide pairs’ annealing temperatures used are shown in Table S1. The PCR was performing using a T100 Thermal Cycler (Bio-Rad, Hercules, CA, USA). The results were analyzed using gel electrophoresis with a GeneRuler 100 bp Plus DNA Ladder (Thermo Fisher Scientific, Vilnius, Lithuania) as a reference.

Every PCR-positive result was confirmed with Sanger sequencing. Briefly, putative positive PCR products were gel-purified using a QIAquick Gel Extraction Kit (QIAGEN, Hilden, Germany) and sequenced using virus-specific oligonucleotides (Table S1). Sequencing was carried out with an ABI PRISM 3500 (Applied Biosystems, Foster City, CA, USA) sequencer using ABI PRISM^®^ BigDye™ Terminator v. 3.1. The sequences were assembled using the SeqMan software v.7.0.0 (DNASTAR Inc., Madison, WI, USA). The sequence origin was determined using the BLAST program [28]. The PCR probe was considered positive only if the virus’ presence was confirmed through sequencing.

### 2.5. Phylogenetic Tree Construction

From the full-genome sequences obtained, RNA-dependent RNA polymerase amino acid sequences were extracted. These sequences, along with their homologs, were aligned in MAFFT v7.3.10 [29] using 1000 cycles of iterative refinement with the FFT-NS-i method and then processed with the TrimAL v1.4. rev 15 program [30] with the default parameters to remove poorly aligned regions. Using these data, maximum-likelihood phylogenetic trees were constructed in the phyML 3.3.20200621 program [31] with 1000 bootstrap replications using the default parameters and default model (LG). The phylogenetic trees obtained were processed in FigTree v.1.4.4. Genomes of the viruses were drawn using a custom Python script. All postprocessing of the images was performed with the GNU Image manipulation program v.2.10.24.

### 2.6. Virus Passages

To assess the ability of rhabdoviruses to replicate in mammalian cells, five cell cultures were used: a human epithelial (HEP-2) cell line, a baby hamster kidney (BHK) cell line, a human embryo kidney (HEK293T) cell line, a porcine embryo kidney (PEK) cell line, and a mouse fibroblast (NIH) cell line. The PEK cell line was maintained in Medium 199: Earle’s Salts (FSASI Chumakov FSC R&D IBP RAS, Moscow, Russia) supplemented with 5% FBS (Gibco, Paisley, UK) and antibiotics (100 U/mL penicillin, 100 µg/mL streptomycin). Other mammalian cell lines were maintained in DMEM (FSASI Chumakov FSC R&D IBP RAS, Moscow, Russia) supplemented with 10% fetal bovine serum (Gibco, Paisley, UK).

For the experiment, cells were seeded in flat-sided cell culture tubes (NUNC, ThermoFisher Scientific, Roskilde, Denmark) and cultivated at 37 °C until a count of 0.5–2 × 10^6^ cells per tube was reached (usually for one or two days). Then, supernatant was discarded and the cells were infected with either 150 µL of IRE/CTVM19 culture supernatant or with 150 µL of culture supernatant from the previous passage. After adsorption under the experimental conditions (either 32 °C or 28 °C) for 30 min, 2 mL of the maintenance medium (for PEK cells) or 5% FBS maintenance medium (for HEP-2, BHK, HEK293T, and NIH cells) was added with 0.1% penicillin/streptomycin. The experimental tubes were incubated for the observation period (7 to 14 days) in a thermostat at 32 °C or 28 °C.

## 3. Results

### 3.1. Viruses Found in the IRE/CTVM19 Cell Line

In the first part of the study, we performed high-throughput sequencing on the supernatant of the uninfected IRE/CTVM19 cell line to reveal the viruses present in the culture. After filtering the reads by quality and length, a total of 2.169 million sequencing reads were obtained. Subsequent data processing allowed us to identify the genome sequences of three previously described rhabdoviruses: Norway mononegavirus 1 (NMV-1), Chimay rhabdovirus (CRV), and IARV. To obtain the full genomes of these viruses from our data, filtered reads were mapped to the known genomes of these rhabdoviruses, downloaded from the GenBank database (Table 1). 

**Figure 1:**
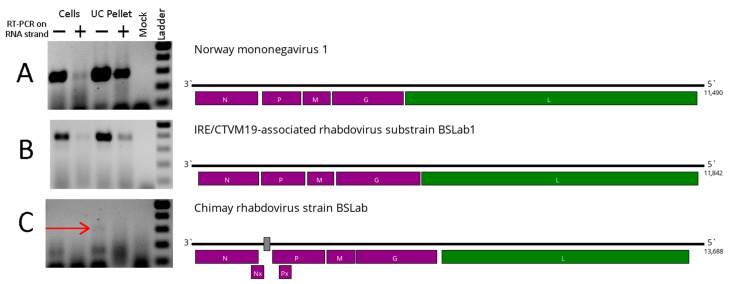
The presence of the rhabdovirus RNA (**left**), as well as the genome scheme (**right**) of all detected viruses: (**A**) Norway mononegavirus 1, (**B**) IRE/CTVM19-associated rhabdovirus, (**C**) Chimay rhabdovirus. On the left: cells—IRE/CTVM19 cells; UC pellet—IRE/CTVM19 supernatant ultracentrifugation pellet; Mock—negative control; + and − mark target viral strand during RT-PCR; Ladder—DNA ladder (100–500) bp range; the red arrow points to the Chimay-rhabdovirus-positive band. On the right: the putative RNA-dependent RNA polymerase is marked in green; undetermined nucleotides in the Chimay rhabdovirus genome are shown as a gray box.

The full coding genomes of NMV-1 and IARV were obtained, as well as the near-complete sequence of the CRV genome (Figure 1). Previously, we reported the presence of IARV (strain BSLab) in the IRE/CTVM19 cell line [18]. In the current work, we also recovered the full IARV genome from the supernatant of the IRE/CTVM19 cell line. To avoid confusion in the naming, the virus variant recovered here was named substrain BSLab1. Despite these variants being separated by 3 years in terms of the passaging history (Figure S1), they are very close and have only 16 substitutions between them (Table 1).

In the case of CRV, the resulting assembly had several regions of the genome with no mapped reads, which was probably due to the much lower presence of CRV reads in the sample (Table 1). Subsequently, we attempted to use raw reads to assemble the full CRV genome; however, one region (166 nt in length) with no mapped reads remained. Attempts to bridge the gap using RT-PCR and subsequent Sanger sequencing failed, leading to the partial CRV genome (Figure 1).

The genomes obtained showed high similarity with previously deposited sequences of the same viruses in the GenBank database (less than 2% nucleotide divergence in the polymerase sequence). The sample contained fairly large amounts of NMV-1 and IARV: 5.55% and 3.78% of the total obtained reads. The abundance of CRV was low: 0.12% of the total obtained reads. 

Using virus-specific oligonucleotides, we tested IRE/CTVM19 cells, as well as the supernatant’s ultracentrifugation pellet, for the presence of the virus RNA. A strand-specific PCR was performed for each sample, in order to distinguish the genome (RT-PCR targeting negative RNA strand) and replicative form (RT-PCR targeting positive RNA strand). For NMV-1 and IARV, both strands could be detected in the cells and ultracentrifugation pellet (Figure 1), suggesting the virus’ presence and active replication in the IRE/CTVM19 cell line. For CRV, only the negative strand of the genome could be found in the ultracentrifugation pellet, and no PCR amplification was detected in the IRE/CTVM19 cells.

### 3.2. Phylogenetic Analysis

**Figure 2:**
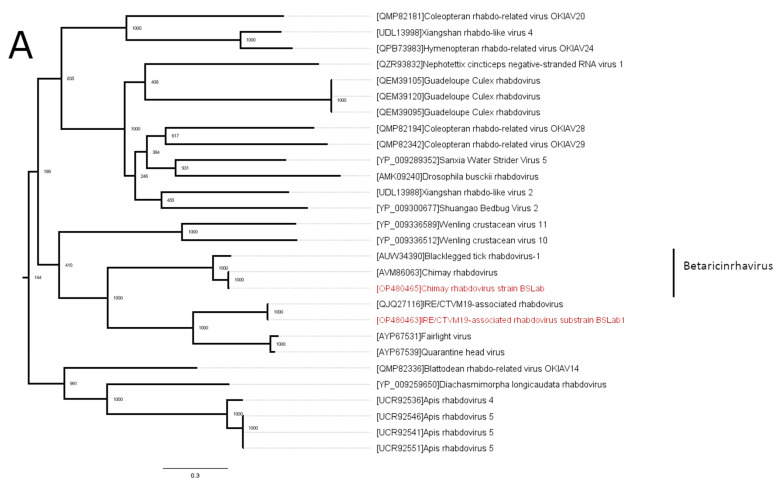

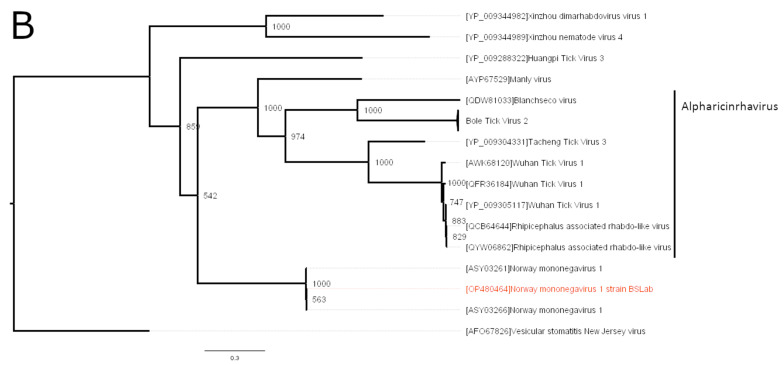
The phylogenetic relationships of the rhabdoviruses found in this study. The analysis was performed using the amino acid sequences of the L protein, with 1000 bootstrap replicates. The scale bar represents the number of amino acid substitutions per site. The viruses discovered in the IRE/CTVM19 cell line are marked in red. (**A**) Midpoint-rooted phylogenetic tree of the IRE/CTVM19-associated rhabdovirus and Chimay rhabdovirus 1. (**B**) The phylogenetic relationships of the Norway mononegavirus. Vesicular stomatitis virus is used as an outgroup.

Phylogenetically, CRV from the IRE/CTVM19 cell line clustered with a previously discovered CRV strain. Along with blacklegged tick rhabdovirus 1, CRV forms the genus *Betaricinrhavirus* (Figure 2A). IARV is close to the genus *Betaricinrhavirus*, forming a monophyletic branch (Figure 2A) together with Fairlight virus and Quarantine Head virus (related to genus *Betaricinrhavirus*, but unclassified in the genus thus far, according to the International Committee on Taxonomy of Viruses (ICTV)). However, IARV is highly divergent from its closest relatives. It shares only a 48% identity with its closest relative (Fairlight virus) in the L protein and 32% in the G protein. Moreover, the most similar virus is Apis rhabdovirus 4 in the N protein sequence (28%), and it is significantly different in terms of the overall genome structure from typical *Betaricinrhavirus* members, such as CRV, because it lacks the Nx and Px open reading frames (Figure 1).

The third virus found in the current study was NMV-1. Phylogenetically, NMV-1 is close to the recently formed genus *Alpharicinrhavirus* (Figure 2B) and is currently considered to be a related unclassified virus.

### 3.3. Passaging Experiment

Tick cell cultures are often used for the isolation and propagation of known and novel pathogens. Afterward, the culture supernatant of tick cells can be used for experiments on mammals and mammalian cell cultures. In such a case, if the viruses revealed were able to multiply in mammalian cell cultures, they could impact the experimental results. Accordingly, we decided to test if these viruses were able to multiply in five mammalian cell cultures: HEP-2, BHK, NIH, HEK293T, and PEK. The results obtained are presented in Table 2.

Three to four passages were performed at temperatures of 32 °C and 28 °C, with monitoring of the virus’ presence in the culture supernatant at 7 and 14 days post-infection using virus-specific oligonucleotides. No virus RNA was detected in the cell cultures cultivated at 32 °C at the second and following passages (Table 2).

CRV was found after the first passage in all tested cell cultures except for BHK. However, no CRV RNA was found in the second and third passages in any of the cell cultures. IARV was found in all tested cultures after the first passage; in HEP-2, BHK, and PEK after the second passage; and in HEP-2 and BHK after the third passage. NMV-1 was detected in all tested cultures after the first passage, in HEP-2 and BHK after the second passage, and in HEP-2 after the third passage. 

However, no virus RNA was detected in the culture supernatant after four passages. These data indicate that it is unlikely that the viruses detected in IRE/CTVM19 cells can replicate in the mammalian cell cultures that were tested. 

## 4. Discussion

In the current work, we used high-throughput sequencing to uncover viruses persisting in an IRE/CTVM19 cell culture. We were able to identify three viruses (CRV, NMV-1, and IARV) and to obtain the full-genome sequences of two of them (NMV-1 and IARV). Phylogenetically, all viruses that we found were close to other tick-infecting rhabdoviruses of the genera *Alpharicinrhavirus* and *Betaricinrhavirus*. CRV is a member of the *Betaricinrhavirus* genus [32,33], detected in Belgium in *I. ricinus* ticks [32]. IARV is close to Fairlight virus and Quarantine Head virus, which are considered to be related to the genus *Betaricinrhavirus*, but are still unclassified [33]. IARV has more than 15% divergence in the N, L, and G proteins, as well as differences in genome organization, compared to the typical *Betaricinrhavirus* members. Thus, it satisfies at least four criteria determined by the ICTV for a new species of the genus *Betaricinrhavirus* [33]. The NMV-1 sequences formed a separate but relatively close branch of the recently formed genus *Alpharicinrhavirus* (Figure 2B). When first discovered, there was doubt about whether NMV-1 was a tick virus [22]. Later, it was found in *I. ricinus* pools from Belgium, and the assumption that NMV-1 was a low-abundance tick virus was made [32]. 

Previously, data have indicated the presence of reovirus virions, iflavivirus, and rhabdovirus RNA in IRE/CTVM19 cells [16,17]. In the current work, we searched for viruses in the ultracentrifuged supernatant of an IRE/CTVM19 cell culture. Here, we found no sequences with similarities to the members of *Reoviridae* or *Iflaviridae*. It is possible that these viruses were not released from the cells; were lost during laboratory maintenance throughout the years; or could persist with relatively low RNA amounts, making them challenging to detect, even with high-throughput sequencing. 

For novel viruses, especially those detected using the high-throughput sequencing approach, it is often difficult to determine whether a virus is incorporated into the host genome without performing additional experiments. Classic endogenous virus elements are incorporated into a host genome and are mostly defective [34,35], either because they were originally inserted as a partial sequence of the virus genome or because they acquired stop-codon mutations after the insertion event [34]. 

We were able to obtain the full-genome sequences of NMV-1 and IARV and obtained a PCR product while targeting the positive strand of RNA. Taken together, our findings imply that these two viruses are likely to be fully functional, with the ability to replicate in the IRE/CTVM19 cell line. 

However, a clear conclusion cannot be made in the case of CRV. Its presence in the sample was low (0.12% of the reads), and our attempts to obtain the full virus genome (including one using Sanger sequencing) were unsuccessful. However, ultracentrifugation was used prior to sequencing to clear the culture supernatant from the cell debris, making it less likely that the host DNA was sequenced. Additionally, we were unable to detect a signal while targeting the positive strand of the RNA. This might be explained by the overall low level of CRV replication in the IRE/CTVM19 cell line; however, it is also possible that two partial CRV sequences were inserted in the IRE/CTVM19 cell line genome. Further work is needed to clarify this.

In the passaging experiments, we tested the ability of the discovered rhabdoviruses to reproduce in several mammalian-derived cell lines: HEP-2, BHK, NIH, PEK, and HEK-293T. No CRV RNA was found in the second, third, or fourth passages in any of the cell cultures. IARV and NMV-1 were detected in some mammalian-derived cell lines cultivated at 28 °C up to the third passage (Table 2), but they were not detected in the fourth passage. This suggests that the discovered rhabdoviruses are unable to reproduce in these cell lines, or that their reproduction level was below our detection limit. However, we cannot rule out the possibility that these viruses are able to reproduce in different cells of vertebrate origin.

Tick cell lines are often used for the isolation of viruses [6]. It is known that the presence of one virus can have an impact on the reproduction of other viruses [36,37,38]. The impacts of the rhabdoviruses described here on the replication of other viruses are yet to be studied. Thus, whenever possible, several tick cell cultures should be used when isolating novel viruses. 

Other tick cell lines are suspected to contain viruses [16,17]. Here, we not only found two previously known viruses in IRE/CTVM19 but also confirmed the presence of a novel virus named IRE/CTVM19-associated rhabdovirus, which we found in our previous work [18]. This confirms that tick cell lines are an easily accessible resource for virus discovery [17] and that a metagenomic approach can both help to characterize tick cell lines and reveal valuable information about tick viruses and their evolution.

## Figures and Tables

**Table 1:** The identities of the viruses found in the IRE/CTVM19 cell culture, with the closest entries from GenBank.

Virus	Percent of the Virus-Containing Reads	Closest Sequence	Nucleotide Identity in Polymerase
Chimay rhabdovirus strain BSLab	0.12%	[MF975531]	99.21%
IRE/CTVM19-associated rhabdovirus substrain BSLab1	3.78%	[MT181988]	99.92%
Norway mononegavirus 1 strain BSLab	5.55%	[MF141072]	98.30%

**Table 2:** Replication of Norway mononegavirus 1, Chimay rhabdovirus, and IRE/CTVM19-associated rhabdovirus in mammalian cell cultures.

Cell Line	Temperature	1st Passage		2nd Passage		3rd Passage		4th Passage
		7 Days	14 Days	7 Days	14 Days	7 Days	14 Days	7 Days
HEP-2	28 °C	I N C	N	I N	I N	I N	-	-
	32 °C	I N C	I N	-	-	-	-	nd
BHK	28 °C	I N	I N	I N	I N	I	-	-
	32 °C	I	I N	-	cd	nd	nd	nd
NIH	28 °C	I N C	I N C	-	-	-	-	nd
	32 °C	-	cd	-	-	-	-	nd
HEK-293T	28 °C	I N C	cd	-	cd	-	-	nd
	32 °C	I N	N	-	-	-	cd	nd
PEK	28 °C	-	I N C	I	-	nd	nd	nd
	32 °C	-	N	-	-	-	-	nd

I—IRE/CTVM19-associated rhabdovirus RNA was detected; N—Norway mononegavirus 1 RNA was detected; C—Chimay rhabdovirus RNA was detected; cd—probe not analyzed due to cell death on 7th day; -—RNA of the viruses not detected; nd—not done.

## Data Availability

The reads obtained were deposited in the sequence read archive database under accession number PRJNA879987.

## Supplementary Materials

The following supporting information can be downloaded at https://www.mdpi.com/article/10.3390/v16040576/s1, Table S1: Oligonucleotide pairs used for the rhabdovirus detection; Figure S1: Passage history of the IRE/CTVM19-associated rhabdovirus strains discussed in this study. Strain used in the current work marked in green.

## References

[1] Bonnet S.I., Pollet T. (2020). Update on the intricate tango between tick microbiomes and tick-borne pathogens. Parasite Immunol..

[2] Gray J., Kahl O., Zintl A. (2021). What do we still need to know about *Ixodes ricinus*?. Ticks Tick. Borne. Dis..

[3] Bogovic P., Strle F. (2015). Tick-borne encephalitis: A review of epidemiology, clinical characteristics, and management. World J. Clin. Cases.

[4] Marques A.R., Strle F., Wormser G.P. (2021). Comparison of lyme disease in the United States and Europe. Emerg. Infect. Dis..

[5] Ruzek D., Av T., Borde J., Chrdle A., Eyer L., Karganova G., Kholodilov I., Kozlovskaya L., Matveev A., Miller A.D. (2019). Tick-borne encephalitis in Europe and Russia: Review of pathogenesis, clinical features, therapy, and vaccines. Antivir. Res..

[6] Bell-Sakyi L., Zweygarth E., Blouin E.F., Gould E.A., Jongejan F. (2007). Tick cell lines: Tools for tick and tick-borne disease research. Trends Parasitol..

[7] Lawrie C.H., Uzcátegui N.Y., Armesto M., Bell-Sakyi L., Gould E.A. (2004). Susceptibility of mosquito and tick cell lines to infection with various flaviviruses. Med. Vet. Entomol..

[8] Mazelier M., Rouxel N., Zumstein M., Mancini R., Bell-Sakyi L., Lozach P. (2016). Uukuniemi virus as a tick-borne virus model. J. Virol..

[9] Bell-Sakyi L., Kohl A., Bente D.A., Fazakerley J.K. (2012). Tick cell lines for study of crimean-congo hemorrhagic fever virus and other arboviruses. Vector-Borne Zoonotic Dis..

[10] Husin N.A., Khoo J.J., Mustika M., Zulkifli S., Bell-Sakyi L., Abubakar S. (2021). Replication kinetics of *Rickettsia raoultii* in tick cell lines. Microorganisms.

[11] Palomar A.M., Premchand-Branker S., Alberdi P., Belova O.A., Moniuszko-Malinowska A., Kahl O., Bell-Sakyi L. (2019). Isolation of known and potentially pathogenic tick-borne microorganisms from European ixodid ticks using tick cell lines. Ticks Tick. Borne. Dis..

[12] Mangia C., Vismarra A., Kramer L., Bell-Sakyi L., Porretta D., Otranto D., Epis S., Grandi G. (2016). Evaluation of the in vitro expression of ATP binding-cassette (ABC) proteins in an *Ixodes ricinus* cell line exposed to ivermectin. Parasit. Vectors.

[13] Belova O.A., Litov A.G., Kholodilov I.S., Kozlovskaya L.I., Bell-Sakyi L., Romanova L.I., Karganova G.G. (2017). Properties of the tick-borne encephalitis virus population during persistent infection of ixodid ticks and tick cell lines. Ticks Tick. Borne. Dis..

[14] Kholodilov I.S., Belova O.A., Morozkin E.S., Litov A.G., Ivannikova A.Y., Makenov M.T., Shchetinin A.M., Aibulatov S.V., Bazarova G.K., Bell-sakyi L. (2021). Geographical and tick-dependent distribution of flavi-like Alongshan and Yanggou tick viruses in Russia. Viruses.

[15] Attoui H., Stirling J.M., Munderloh U.G., Burroughs F., Brookes S.M., Burroughs J.N., Micco P., Mertens P.P.C., Lamballerie X. (2001). Complete sequence characterization of the genome of the St Croix River virus, a new orbivirus isolated from cells of *Ixodes scapularis*. J. Gen. Virol..

[16] Alberdi M.P., Dalby M.J., Rodriguez-Andres J., Fazakerley J.K., Kohl A., Bell-Sakyi L. (2012). Detection and identification of putative bacterial endosymbionts and endogenous viruses in tick cell lines. Ticks Tick. Borne. Dis..

[17] Bell-Sakyi L., Attoui H. (2016). Virus discovery using tick cell lines. Evol. Bioinforma..

[18] Kholodilov I.S., Litov A.G., Klimentov A.S., Belova O.A., Polienko A.E., Nikitin N.A., Shchetinin A.M., Ivannikova A.Y., Bell-sakyi L., Yakovlev A.S. (2020). Isolation and characterisation of Alongshan virus in Russia. Viruses.

[19] Li C., Shi M., Tian J., Lin X., Kang Y., Chen L., Qin X., Xu J., Holmes E.C., Zhang Y. (2015). Unprecedented genomic diversity of RNA viruses in arthropods reveals the ancestry of negative-sense RNA viruses. Elife.

[20] Shi M., Lin X., Tian J., Chen L., Chen X., Li C., Qin X., Li J., Cao J., Eden J. (2016). Redefining the invertebrate RNA virosphere. Nature.

[21] Harvey E., Rose K., Eden J., Lo N., Abeyasuriya T., Shi M., Doggett S.L., Holmes E.C. (2019). Extensive diversity of RNA viruses in Australian ticks. J. Virol..

[22] Pettersson J.H., Shi M., Bohlin J., Eldhol V., Brynildsrud O.B., Paulsen K.M., Andreassen Å., Holmes E.C. (2017). Characterizing the virome of *Ixodes ricinus* ticks from northern Europe. Sci. Rep..

[23] Lo C.C., Chain P.S.G. (2014). Rapid evaluation and quality control of next generation sequencing data with FaQCs. BMC Bioinform..

[24] Bankevich A., Nurk S., Antipov D., Gurevich A.A., Dvorkin M., Kulikov A.S., Lesin V.M., Nikolenko S.I., Pham S., Prjibelski A.D. (2012). SPAdes: A new genome assembly algorithm and its applications to single-cell sequencing. J. Comput. Biol..

[25] Bolger A.M., Lohse M., Usadel B. (2014). Trimmomatic: A flexible trimmer for Illumina sequence data. Bioinformatics.

[26] Okonechnikov K., Golosova O., Fursov M., UGENE team (2012). Unipro UGENE: A unified bioinformatics toolkit. Bioinformatics.

[27] Langmead B., Salzberg S. (2013). Fast gapped-read alignment with Bowtie 2. Nat. Methods.

[28] Johnson M., Zaretskaya I., Raytselis Y., Merezhuk Y., Mcginnis S., Madden T.L. (2008). NCBI BLAST: A better web interface. Nucleic Acids Res..

[29] Katoh K., Standley D. (2013). MAFFT multiple sequence alignment software version 7: Improvements in performance and usability. Mol. Biol. Evol..

[30] Capella-Gutierrez S., Silla-Martinez J.M., Gabaldon T. (2009). trimAl: A tool for automated alignment trimming in large-scale phylogenetic analyses. Bioinformatics.

[31] Guindon S., Gascuel O. (2003). A simple, fast, and accurate algorithm to estimate large phylogenies by maximum likelihood. Syst. Biol..

[32] Vanmechelen B., Merino M., Vergote V., Laenen L., Thijssen M., Martí-carreras J., Claerebout E., Maes P. (2021). Exploration of the *Ixodes ricinus* virosphere unveils an extensive virus diversity including novel coltiviruses and other reoviruses. Virus Evol..

[33] Walker P., Freitas-Astúa J., Walker P.J., Astúa J.F.-, Bejerman N., Blasdell K.R., Breyta R., Dietzgen R.G. (2022). ICTV virus taxonomy profile: *Rhabdoviridae* 2022. J. Gen. Virol..

[34] Holmes E.C. (2011). The evolution of endogenous viral elements. CHOM.

[35] Katzourakis A., Gifford R.J. (2010). Endogenous viral elements in animal genomes. PLoS Genet..

[36] Abrao E.P., Lopes da Fonseca B. (2016). Infection of mosquito cells (C6/36) by Dengue-2 virus interferes with subsequent infection by Yellow Fever Virus. Vector-Borne Zoonotic Dis..

[37] Kuwata R., Isawa H., Hoshino K., Sasaki T., Kobayashi M., Maeda K., Sawabe K. (2015). Analysis of mosquito-borne flavivirus superinfection in *Culex tritaeniorhynchus* (Diptera: Culicidae) cells persistently infected with culex flavivirus (*Flaviviridae*). J. Med. Entomol..

[38] Patterson E.I., Kautz T.F., Contreras-gutierrez M.A., Guzman H., Tesh R.B., Hughes G.L., Forrester N.L. (2021). Negeviruses reduce replication of alphaviruses during coinfection. J. Virol..

