# Supplementary material for: High-Throughput Sequencing Reveals Three Rhabdoviruses Persisting in the IRE/CTVM19 Cell Line
Source: Viruses. 2024 Apr 9;16(4):576. doi: 10.3390/v16040576 (PMC11054507; doi:10.3390/v16040576)

Table S1. Oligonucleotide pairs used for the rhabdovirus detection.

Virus	Name	Sequence	Temperature
Chimay rhabdovirus	Chimay_L_F	5`-TCTTGAGCTTTGGTGGTCCG-3`	55°C
	Chimay_L_R	5`-CATCTTGTACACCCGCCCTT-3`	
Norway mononegavirus 1	Norway_L_F2	5`-ACACACTCCATCCTTTTTGT-3`	50°C
	Norway_L_R2	5`-ACTACTCAAGCATCGGAATC-3`	
IRE/CTVM19-associated rhabdovirus	Rhabdo_Miass_L_1F	5`-GGGTTTGTGGTTAATTTGTC-3`	50°C
	Rhabdo_Miass_L_1R	5`-AGTGAGGACTGGATAAAAGA-3`	

Figure S1. Passage history of the IRE/CTVM19-associated rhabdovirus strains discussed in the study. Strain used in the current work marked in green.

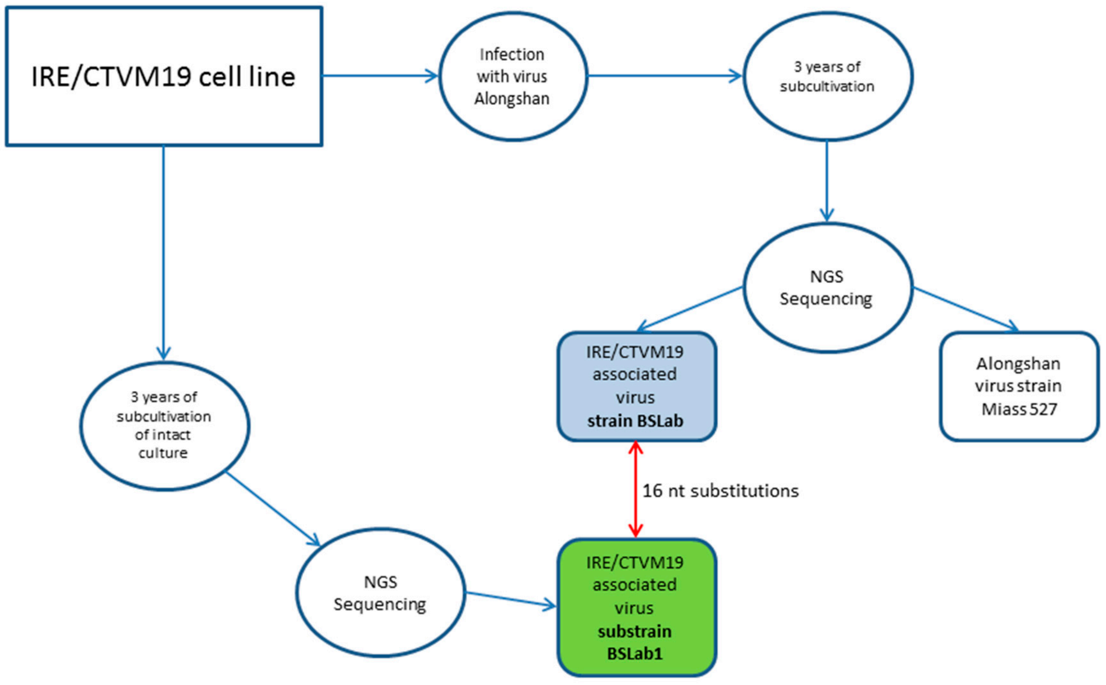

Supplement: Supplementary file 1 [file viruses-16-00576-s001.zip › viruses-2940563-supplementary.pdf]
